# Genome-Wide Identification of the *PHR* Gene Family in Six Cucurbitaceae Species and Its Expression Analysis in *Cucurbita moschata*

**DOI:** 10.3390/plants14101443

**Published:** 2025-05-12

**Authors:** Ying Ni, Kailing Xie, Minghui Shi, Hanchen Shan, Wenxiang Wu, Weiwei Wang, Beijiu Cheng, Xiaoyu Li

**Affiliations:** 1 Key Laboratory of Crop Stress Resistance and High Quality Biology of Anhui Province, Anhui Agricultural University, Hefei 230036, China; 15215517537@163.com (Y.N.); 15056907002@163.com (K.X.); 18905619514@163.com (M.S.); shanhc2022@163.com (H.S.); 15255159313@163.com (W.W.); 2 College of Biology and Food Engineering, Suzhou University, Suzhou 234000, China; wangweiwei@ahszu.edu.cn

**Keywords:** phosphate-starvation response, genome-wide analysis, transcription factor, Cucurbitaceae, *Cucurbita moschata*

## Abstract

Phosphorus, as an essential nutrient, plays an important role in plant growth and development. Although Phosphate Starvation Response 1 (PHR1) or PHR1-like have been recognized as central regulators of phosphorus (Pi) homeostasis in several plants, they have not been systematically studied in Cucurbitaceae. In this study, 11, 10, 8, 12, 12, and 22 *PHR* genes were identified in cucumber, melon, bottle gourd, watermelon, wax gourd, and pumpkin, respectively, by genome-wide analysis. Phylogenetic analysis showed that the Cucurbitaceae *PHR* genes were divided into seven distinct subfamilies. These genes were further phylogenetically analyzed for their chromosomal localization, gene structure, protein structure, and synteny. Genomic homology analysis showed that many *PHR* genes existed in the corresponding homology blocks of six Cucurbitaceae species. qRT-PCR analysis showed that the *CmoPHR* genes exhibited differential expression under different concentrations of phosphate treatment. Transcriptional self-activation assays showed that CmoPHR2, CmoPHR9, CmoPHR16, and CmoPHR17 proteins had transcriptional self-activating activity. The results of this study provide a basis for the further cloning and functional validation of genes related to the phosphate regulatory network in pumpkin.

## 1. Introduction

Phosphorus is a major limiting nutrient that affects crop growth. It is the main component of phospholipids, ATP, nucleic acids, pyrophosphates, and other key molecules and participates in important physiological and biochemical processes such as energy metabolism, redox reactions, protein activation, and photosynthesis [1,2,3,4]. However, it is one of the most difficult elements to obtain [5,6]. This difficulty stems from its low solubility, low mobility, and poor ability to bind to organic compounds. Approximately 20–80% of the phosphorus in soils exists mainly as phytic acid in organic matter [7,8]. Moreover, in acidic soils, phosphate forms insoluble precipitates with aluminum and iron, whereas in alkaline soils, phosphorus reacts with calcium and magnesium ions [9,10], resulting in low levels of available phosphorus in the soil, which, in turn, causes phosphorus-deficiency stress in plants [11]. Phosphorus deficiency frequently limits plant growth in both agroecosystems and natural environments [12]. It has been estimated that plants are thought to be able to use less than 20% of the phosphorus from the soil, even when appropriate levels of phosphorus fertilizer are applied [13]. Crop production requires the application of excessive phosphorus fertilizers to replenish the effective phosphorus in the soil; however, this process can seriously pollute the environment [14,15]. Therefore, in order to achieve sustainable production in Pi-deficient soils, it is necessary to investigate and study the molecular basis for the optimal consumption of phosphorus.

Under low-phosphorus or phosphorus-deficiency stress, plants undergo changes in their morphology and physiological and biochemical activities, which are collectively referred to as phosphorus-starvation responses (PSRs) [3]. These include severe growth inhibition, an increased root–crown ratio, a greater number of lateral roots, phosphorus-starvation-induced (PSI) gene expression, and starch and anthocyanin accumulation [2,16]. Maintaining phosphorus homeostasis in plants is primarily achieved through two strategies: one is to conserve the internal phosphorus utilization efficiency, and the second is to enhance the capacity to acquire or to absorb external phosphorus sources [17]. Ways to economize on phosphorus use include reducing growth rates, remobilizing phosphorus, changing the carbon metabolism (skipping steps that require phosphorus), and using alternative respiratory pathways; Ways to enhance phosphorus acquisition or absorption include changing the root structure, enhancing the expression and activity of high-affinity phosphorus transporters, inducing and secreting acid phosphatases (APases) and organic acids, and promoting *PSI* gene expression, etc. [16,17]. In addition, plants can obtain and utilize phosphorus indirectly by interacting with microorganisms [18,19].

To adapt to low-phosphorus environments, plants have developed a complex gene regulatory network, of which the most important members related to phosphate uptake and transport include PHR1 (Phosphate Starvation Response 1), IPS1 (Phosphate Starvation 1), miR399 (microRNA399), PHO2 (Phosphate 2), and PT (Phosphate Transporter) [20,21]. In this complex regulatory network, the PHR protein is an important transcription factor in the plant phosphorus regulatory network and plays a key role in phosphate-starvation-induced signaling and regulation. PHR1 transcription factors belong to the MYB-CC gene family and contain the MYB domain and convoluted helix (CC) domain [2], which regulate their expression by binding to the P1BS element (GNATATNC) in the promoter region of the *PSI* gene [22]. The *CrPSR1* gene of *Chlamydomonas rheinensis* was the first regulatory factor identified in response to low-phosphorus stress [23,24]. The *AtPHR1* gene, identified in *Arabidopsis thaliana*, is a homologue of *CrPSR1*. *AtPHR1* loss of function in *atphr1* mutants was found to cause an impaired phosphorus-starvation response, including the reduced expression of *PSI* genes (*AtIPS1*, *At4*, *Mt4/TPSI1*, *AtPT1*, *AtACP5*, etc.), decreased biomass, and reduced anthocyanin, Pi, glucose, fructose, and starch contents [22,25]. Similarly, *OsPHR2*, a homologous gene in rice (*Oryza sativa* L.), is considered a major regulator of phosphorus signaling in rice, and overexpression of *OsPHR2* induces changes such as excessive phosphorus accumulation in rice leaves, the upregulation of *PSI* gene expression, and increases in the root length, root–crown ratio, and root–hair density [26,27]. It has been found that *TaPHR1* is involved in phosphorus signal transduction in wheat (*Triticum aestivum* L.), and the overexpression of *TaPHR1* in wheat upregulates a subgroup of phosphate-starvation response genes, stimulates lateral branches, and improves phosphorus absorption [28].

However, most of the current research focuses on model plants and staple crops, while the systematic identification, evolutionary relationship, and functional differentiation of *PHR* genes in Cucurbitaceae, a family containing cucumber, melon, watermelon, and other economically important crops, still lacks comprehensive analysis. Cucumber (*Cucumis sativus* L.), melon (*Cucumis melo* L.), watermelon (*Citrullus lanatus* L.), wax gourd (*Benincasa hispida* L.), bottle gourd (*Lagenaria siceraria* L.), pumpkin (*Cucurbita moschata* L.), and other significant crops are members of the Cucurbitaceae family. These crops exhibit unique traits, like melon fruit and various flower sex types. Cucurbitaceae generally have well-developed root systems and efficient phosphorus activation. For example, pumpkin rootstocks can significantly improve the phosphorus utilization efficiency of scion crops [29,30], but whether the molecular mechanism is related to the functional diversity of the PHR family remains unclear.

In this study, we performed a comprehensive analysis of *PHR* genes in six Cucurbitaceae species: cucumber, melon, watermelon, winter melon, wax gourd, bottle gourd, and pumpkin. We investigated the basic properties, gene structure, chromosomal localization, and cis-acting elements of these genes. In addition, we combined low-phosphorus and high-phosphorus stress experiments to clarify the tissue-specific expression patterns and stress-response characteristics of pumpkin *CmoPHR* genes using qRT-PCR. This study is the first to carry out cross-species comparative genomic analysis of the *PHR* family in Cucurbitaceae and focuses on pumpkin to elucidate its expression regulation mechanism, which provides a theoretical basis for analyzing the molecular basis of efficient phosphorus utilization and genetic improvement in Cucurbitaceae plants.

## 2. Results

### 2.1. Identification of PHR Genes in Cucurbitaceae

In this study, the following six Cucurbitaceae species were selected for analysis: cucumber, melon, bottle gourd, watermelon, wax gourd, and pumpkin. TBtools software (v2.149) was used to compare and analyze the AtPHR, OsPHR, and ZmPHR proteins with the PHR proteins in the six Cucurbitaceae species. A total of 75 PHR proteins were identified in the Cucurbitaceae family. Genomic annotation revealed distinct PHR family sizes across the Cucurbitaceae species: cucumber (*Cucumis sativus*; 11), melon (*Cucumis melo*; 10), bottle gourd (*Lagenaria siceraria*; 8), watermelon (*Citrullus lanatus*; 12), wax gourd (*Benincasa hispida*; 12), and pumpkin (*Cucurbita moschata*; 22). Comprehensive molecular profiles of all 75 *PHR* genes, including their chromosomal localization, CDS length, protein parameters (amino acid count, molecular weight, isoelectric point), and exon organization, are cataloged in Table 1. Notably, *LsiPHR4* exhibited the longest CDS (2238 bp) and encoded the longest polypeptide (745 aa), whereas *CmoPHR1* exhibited the shortest CDS (666 bp) and encoded the shortest polypeptide (221 aa). Predicted pI values indicated alkaline properties in 37 proteins (pI > 7), contrasting with acidic isoforms (pI < 7). Molecular masses spanned 25.51–83.31 kDa, reflecting substantial structural diversity within the family.

### 2.2. Evolutionary Analysis of the PHR Gene Family in Cucurbitaceae

**Figure 1:**
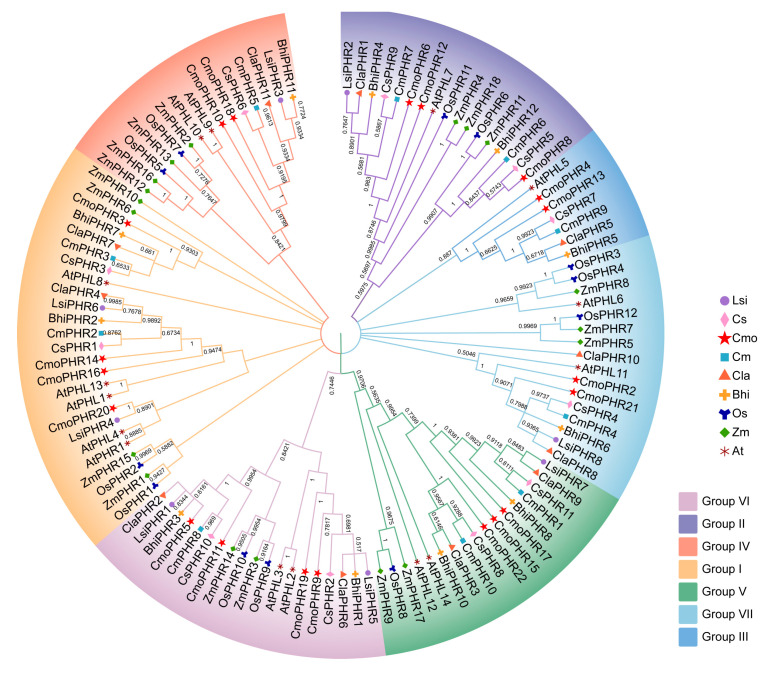
Phylogenetic relationships of PHR proteins from cucumber (Cs), melon (Cm), bottle gourd (Lsi), watermelon (Cla), wax gourd (Bhi), pumpkin (Cmo), Arabidopsis (At), rice (Os), and maize (Zm). All PHR proteins in the nine species were clustered into seven groups, represented by different colors for Groups I to VII.

To delineate the evolutionary relationship of PHR homologs, a neighbor-joining phylogenetic tree was reconstructed using MEGA11 using 120 orthologous sequences comprising 15 Arabidopsis, 18 maize (*Zea mays* L.), 12 rice, and 75 Cucurbitaceae representatives across six analyzed species. As shown in Figure 1, these proteins were divided into seven distinct groups based on their gene structures: Group I: 25 *PHR* members; Group II: 17 *PHR* members; Group III: 7 *PHR* members; Group IV: 15 *PHR* members; Group V: 17 *PHR* members; Group VI: 19 *PHR* members; and Group VII: 16 *PHR* members. A close evolutionary relationship was found between cucumber and pumpkin, watermelon and wax gourd, and bottle gourd and watermelon.

### 2.3. Chromosomal Distribution Heterogeneity of Cucurbitaceae PHR Genes

**Figure 2:**
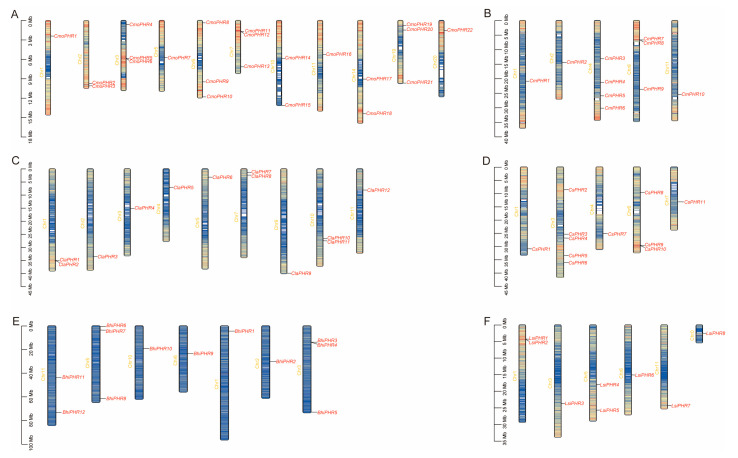
Localization of *PHR* genes on chromosomes of six Cucurbitaceae species. (**A**) Localization of the *CmoPHR* genes on pumpkin chromosomes. (**B**) Localization of the *CmPHR* genes on melon chromosomes. (**C**) Localization of the *ClaPHR* genes on watermelon chromosomes. (**D**) Localization of the *CsPHR* genes on cucumber chromosomes. (**E**) Localization of the *BhiPHR* genes on the wax gourd chromosomes. (**F**) Localization of the *LsiPHR* genes on bottle gourd chromosomes. The chromosome numbers are labelled on the left side of each chromosome. The left scale represents the length of the chromosomes, and the scale is expressed in megabases (Mb). The different colors represented gene density, which gradually increased from blue to red.

Chromosomal mapping using TBtools revealed a non-random genomic distribution of the 75 PHR loci across the six Cucurbitaceae species (Figure 2). The chromosomal distribution patterns exhibited substantial interspecific divergence. The *PHR* genes in pumpkin were distributed across eleven chromosomes, with three *PHR*s located on chromosomes 3, 6, and 19 (Figure 2A). In melon, the *PHR* genes were found on five chromosomes, notably with four *PHR*s residing on chromosome 4 (Figure 2B). Watermelon exhibited *PHR* localization on nine chromosomes, featuring two *PHR*s located on chromosomes 1, 7, and 10 (Figure 2C). Cucumber possessed *PHR* genes distributed across five chromosomes, with a concentration of five *PHR*s present on chromosome 3 (Figure 2D). In the case of wax gourd, *PHR* genes were identified on seven chromosomes, with three located on chromosomes 3 and 5 (Figure 2E). Bottle gourd demonstrated *PHR* localization on six chromosomes, comprising three *PHR*s situated on chromosomes 1 and 5 (Figure 2F). This interspecific heterogeneity suggests that differential chromosomal rearrangement events shaped *PHR* family expansion during the Cucurbitaceae radiation.

### 2.4. Gene Structures, Protein Motifs, and Conserved Domains of Cucurbitaceae PHR Genes

**Figure 3:**
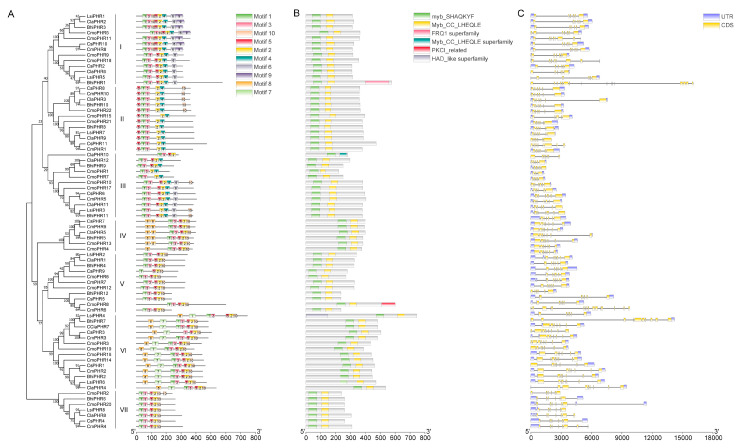
Conserved motifs, domains, and gene structures of PHRs. (**A**) Conserved motifs of PHR proteins. The distribution of these motifs was identified using MEME, and boxes of different colors and numbers represent different motifs. The consensus sequences and amino acid lengths of these motifs are listed. The phylogenetic tree was clustered into seven subgroups ranging from I to VII. (**B**) Conserved domains of the *PHR* genes. Different colors represent different domains. (**C**) Structure of the *PHR* genes. The purple color represents untranslated regions, and yellow represents coding sequences. The lengths of these genes are listed.

Conserved-motif profiling of PHR proteins across the six Cucurbitaceae species was performed via the MEME website. The number of motifs in Groups I, IV, and VI was significantly higher than that in Groups II, III, V, and VII (Figure 3A). Interestingly, Groups IV and VII contained only motifs 1, 3, 5, 2, and 10. Notably, all *PHR* families had motifs 1, 3, and 5. These findings suggest that motifs 1, 3, and 5 are highly conserved and might play an important role in the *PHR* gene family. Moreover, as shown in the phylogenetic tree, PHR proteins exhibited a similar motif composition, with close evolutionary relationships. Domain conservation analysis further highlighted functional constraints through preserved Myb_DNA-binding (PF00249) and Myb_CC_LHEQLE (PF14379) domains across orthologs. As shown in Figure 3B, each PHR protein contained Myb and Myb_CC_LHEQLE domains, which are the major structural domains of PHR proteins, and some proteins contained other domains. For example, BhiPHR1 also contained an FRQ1-superfamily domain, CmoPHR8 also contained a PKCI-related domain, and LsiPHR4 also contained an HAD-like-superfamily domain. Comparative exon–intron structural dissection elucidated evolutionary trajectories within the gene family, with TBtools-based annotations delineating the genomic architecture, including coding sequences (CDSs), untranslated regions (UTRs), and intronic elements. As shown in Figure 3C, *CmoPHR* family members contained 4–16 exons, with an average of 7. *CmoPHR1* contained the fewest exons (4), and *CmoPHR8* contained the most exons (16). Interestingly, *CmoPHR8* did not contain the 5′ and 3′ UTR regions. Members of the *CsPHR* family contained 6–8 exons, *CmPHR* family members contained 6–8 exons, and *LsiPHR* family members contained 6–11 exons, with *LsiPHR4* containing 11 exons and only the 3′-UTR region. *ClaPHR* family members contained 5–9 exons, and *BhiPHR* family members contained 6–17 exons, with *BhiPHR1* containing 17 exons but having no 5′ or 3′ UTR regions.

### 2.5. Cis-Regulatory Element Profiling in Cucurbitaceae PHR Promoters

**Figure 4:**
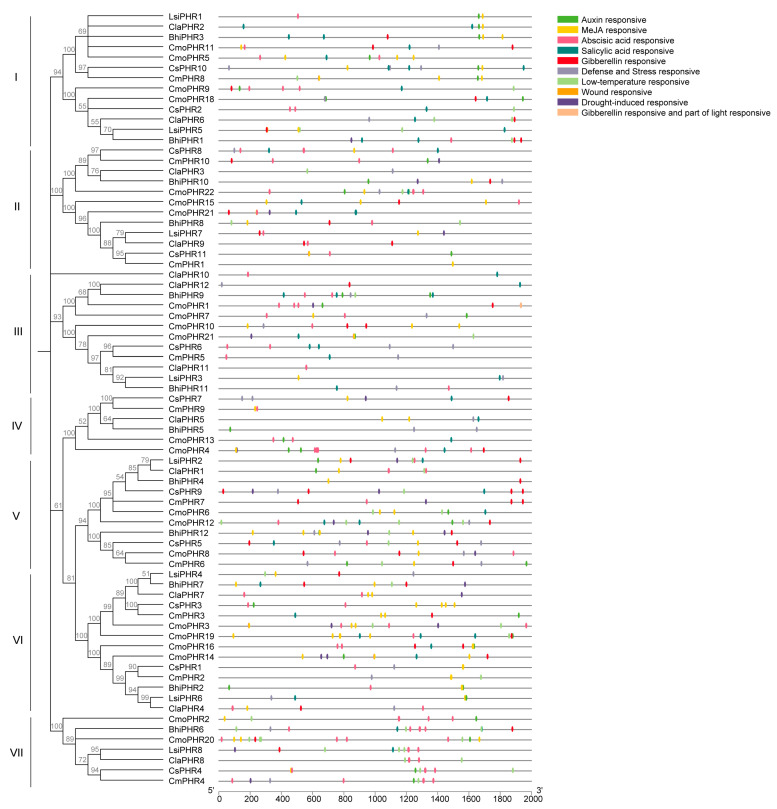
Cis-regulatory element analysis of the *PHR* family members in Cucurbitaceae. Colored boxes indicate different cis-elements in the promoters of the genes. The ruler below represents the length of the promoter. The phylogenetic tree was clustered into seven subgroups ranging from I to VII.

Promoter cis-element mining via PlantCARE revealed 10 functional categories associated with abiotic stress adaptation in Cucurbitaceae PHR genes (Figure 4): phytohormone-responsive modules (auxin, MeJA, ABA, SA, GA), stress-signaling motifs (defense, low-temperature), and developmental regulators. Quantitative interspecific divergence was observed, with pumpkin harboring the highest element density (234), followed by wax gourd (94), melon/watermelon (61 each), and bottle gourd (54). Notably, among these responsive elements, MeJA-responsive elements (CGTCA motif and TGACG motif) were the most numerous, with 182, followed by abscisic acid-responsive elements (ABRE) with 133, whereas wound-responsive elements (WUN-motif) had the lowest frequency of occurrence with only one. This cis-regulatory element implicates *PHR* genes in hormone-stress cross-talk and developmental plasticity under environmental challenges.

### 2.6. Duplication Events in PHR Genes Among Six Cucurbitaceae

**Figure 5:**
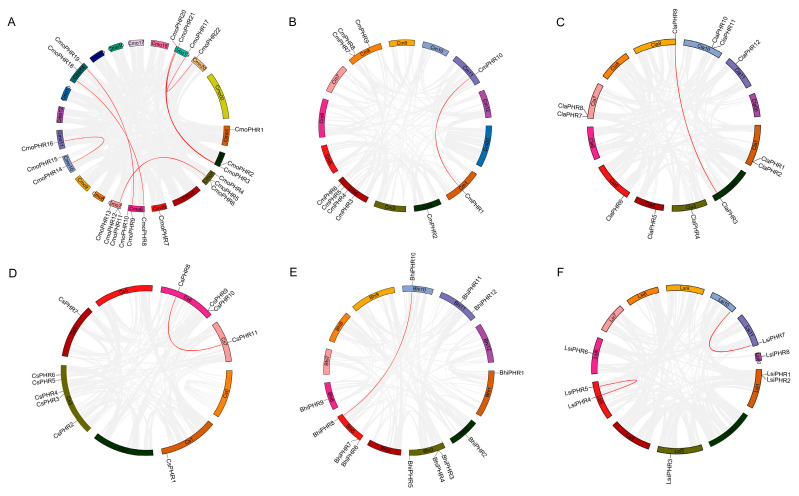
Synteny of the six Cucurbitaceae plant *PHR* genes. (**A**) Synteny of *CmoPHRs* in pumpkin. Cmo1-20 represent pumpkin chromosomes 1–20; (**B**) Synteny of *CmPHRs* in melon. Cm1-12 represent melon chromosomes; (**C**) Synteny of *ClaPHRs* in watermelon. Cla1-11 represent watermelon chromosomes; (**D**) Synteny of *CsPHRs* in cucumber. Cs1-7 represent cucumber chromosomes; (**E**) Synteny of *BhiPHRs* in wax gourd. Bhi1-12 represent wax gourd chromosomes; (**F**) Synteny of *LsiPHRs* in bottle gourd. Lsi1-11 represent bottle gourd chromosomes. All the chromosomes are indicated by colored boxes. Gray lines represent genome-wide collinear genes, whereas red lines represent collinear *PHR* genes.

Based on a comprehensive whole-genome analysis of the six Cucurbitaceae species, pumpkin contains the most putative duplicated gene pairs (*CmoPHR2*/*CmoPHR21*, *CmoPHR3*/*CmoPHR20*, *CmoPHR5*/*CmoPHR11*, *CmoPHR8*/*CmoCh14G004690.1*, *CmoPHR9*/*CmoPHR19*, *CmoPHR14*/*CmoPHR16*, and *CmoPHR17*/*CmoPHR22*), followed by bottle gourd, which contained two gene pairs (*LsiPHR4*/*LsiPHR5*, *LsiPHR7*/*LSI10G015090.01*). The other four species had only one pair (*CmPHR1*/*CmPHR10*, *ClaPHR3*/*ClaPHR9*, *CsPHR8*/*CsPHR11*, *BhiPHR8*/*BhiPHR10*, and *ZmPHR12*/*ZmPHR16*) (Figure 5).

**Figure 6:**
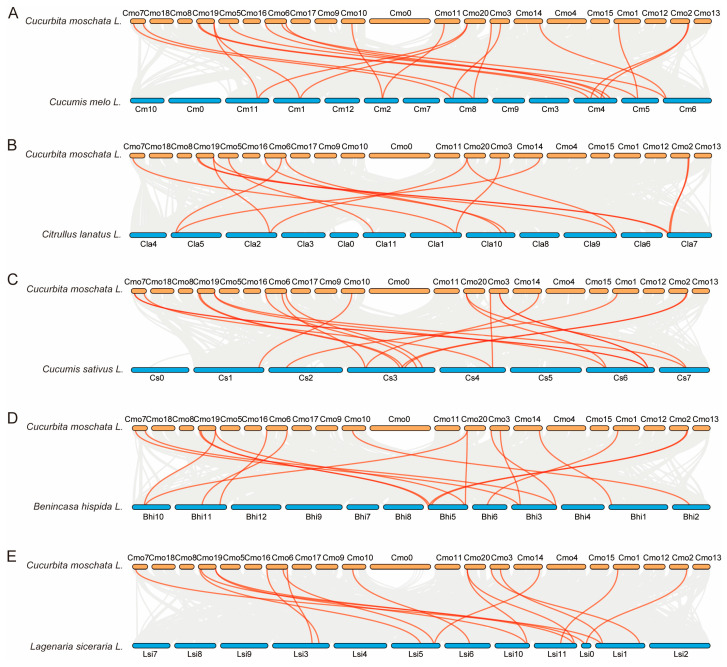
Synteny of *PHR* genes in Cucurbitaceae. (**A**) Synteny of *PHR* between pumpkin and melon. Cmo1-20 represent pumpkin chromosomes, Cm1-12 represent melon chromosomes. (**B**) Synteny of *PHR* between pumpkin and watermelon. Cmo1-20 represent pumpkin chromosomes, Cla1-11 represent watermelon chromosomes. (**C**) Synteny of *PHR* between pumpkin and cucumber. Cmo1-20 represent pumpkin chromosomes, Cs1-7 represent cucumber chromosomes. (**D**) Synteny of *PHR* between pumpkin and wax gourd. Cmo1-20 represent pumpkin chromosomes, Bhi1-12 represent wax gourd chromosomes. (**E**) Synteny of *PHR* between pumpkin and bottle gourd. Cmo1-20 represent pumpkin chromosomes, Lsi1-11 represent bottle gourd chromosomes. Colored boxes indicated chromosomes. Gray lines represent genome-wide collinear genes, whereas red lines represent collinear *PHR* genes.

To better understand the evolutionary history of the *PHR* gene family, we established a putative homologous link between all the *PHR* genes in pumpkin and five other Cucurbitaceae plants (Figure 6). Homologous gene pairs are shown in Table 2. We identified 15 homologous gene pairs between pumpkin and melon, 14 homologous gene pairs between pumpkin and watermelon, 18 homologous gene pairs between pumpkin and cucumber, 17 pairs of homologous genes between pumpkin and wax gourd, and 11 pairs of homozygous genes between pumpkin and bottle gourd. The observed disparity in paralog abundance between pumpkin and the other Cucurbitaceae species likely stems from differential retention rates of duplicated genes during post-WGD (whole-genome duplication) evolution. Notably, among the 22 putative paralogs identified in pumpkin, *CmoPHR15* and *CmoPHR17* were uniquely excluded from all the syntenic blocks, suggesting lineage-specific gene-loss events distinct from the 20 conserved duplicated pairs.

### 2.7. Expression Profiling of CmoPHR Genes in Pumpkin Under Different Pi Conditions

**Figure 7:**
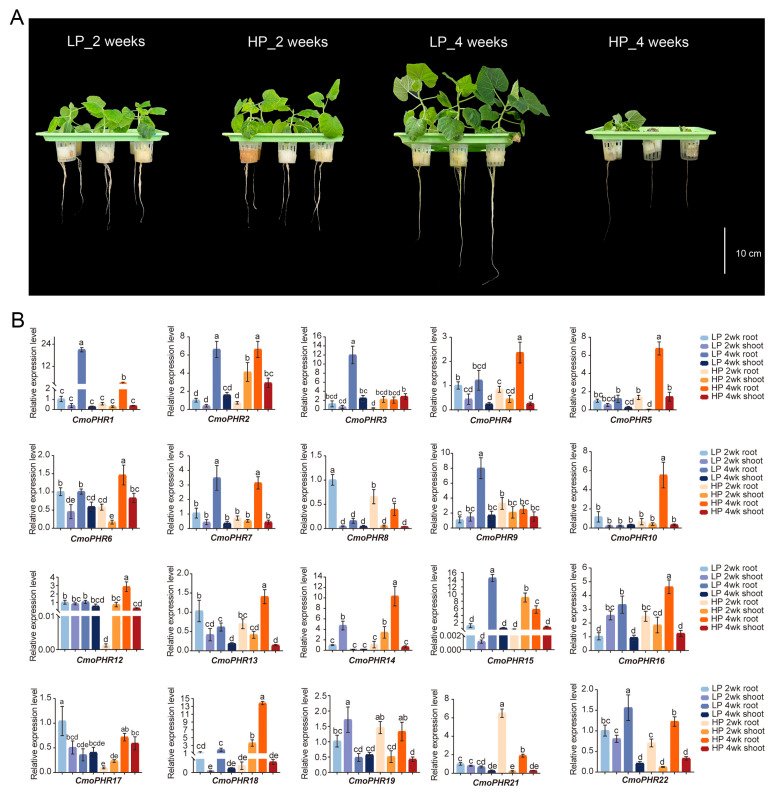
Expression patterns of *CmoPHR* genes in pumpkin in response to Pi stress. (**A**) Pumpkin seedlings at 2 and 4 weeks under low- and high-Pi conditions. The scale is 10 cm. (**B**) Expression levels of *CmoPHR* genes at 2 and 4 weeks under low- and high-Pi conditions, respectively. The standard deviation was obtained from three biological replicates. Different letters (a–e) indicated the degree of significant difference (analyzed by ANOVA and Duncan’s multiple range tests; *p* < 0.05).

Because *PHR* genes are phosphate-starvation-responsive factors, the expression profile of *CmoPHR* genes in pumpkin under Pi treatment (0.05 mM and 2 mM) was further investigated. As shown in Figure 5A, pumpkin plants treated with low-Pi (0.05 mM) for four weeks grew better than those treated for two weeks. With an increase in time, the growth of pumpkin plants under high-Pi (2 mM) treatment for four weeks was worse than that of plants treated for two weeks, and the leaves began to die. The expression of 22 *CmoPHR* genes in the roots and shoots showed that all the *CmoPHR* genes were affected under the low- and high-Pi treatments, except that *CmoPHR11* and *CmoPHR20* were not detected (Figure 7). The expression of *CmoPHR1*, *CmoPHR3*, *CmoPHR9*, and *CmoPHR15* was significantly upregulated in roots treated with low-Pi for 4 weeks, whereas the expression of *CmoPHR4*, *CmoPHR5*, *CmoPHR6*, *CmoPHR10*, *CmoPHR12*, *CmoPHR13*, *CmoPHR14*, *CmoPHR16*, and *CmoPHR18* was significantly upregulated in roots treated with high-Pi for 4 weeks. *CmoPHR2*, *CmoPHR7*, and *CmoPHR22* exhibited upregulation in both low-Pi- and high-Pi-treated roots at 4 weeks post-treatment. *CmoPHR8* and *CmoPHR17* showed induction exclusively under low-Pi at 2 weeks post-treatment. Furthermore, *CmoPHR21* displayed acute upregulation during the initial high-Pi exposure for two weeks. Notably, all 20 *CmoPHR* genes maintained basal expression levels in leaf tissues across all the treatments, indicating organ-specific regulatory divergence.

### 2.8. Transactivation Assay for CmoPHR Proteins

**Figure 8:**
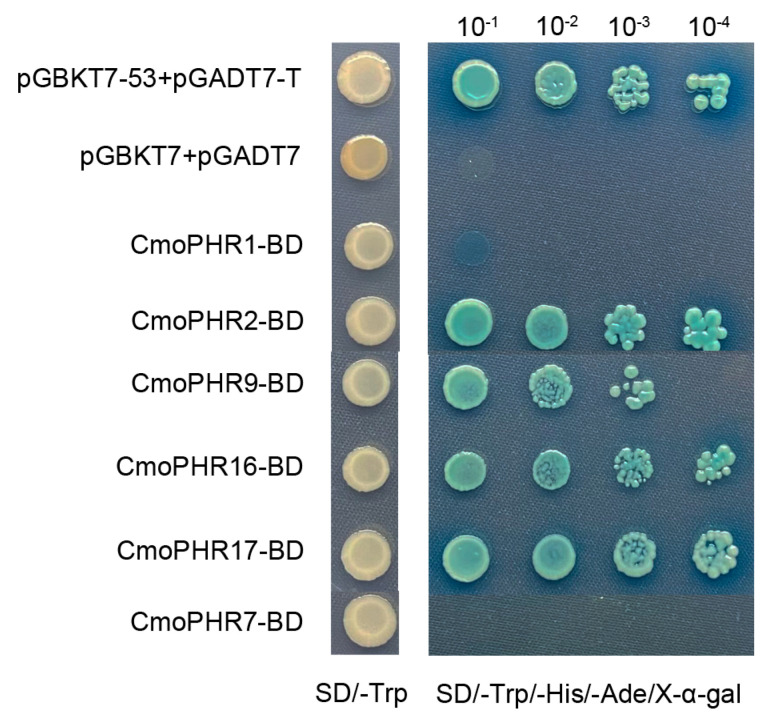
Transactivation assays of four CmoPHR proteins in Y1HGold yeast cells. Transformed yeast cells were grown on SD/-Trp medium or SD/-Trp/-His/-Ade medium. The vector pGBKT7-53+pGADT7-T was used as the positive control, while the empty vector pGBKT7+pGADT7 was used as the negative control. The experiment was repeated three times.

Transcription factors possess distinct DNA-binding domains (DBDs) and transcription activation domains (ADs). The DBD is responsible for recognizing and binding to specific DNA sequences (e.g., promoter or enhancer regions), while the AD initiates or enhances transcription through interactions with other transcription-related proteins, and its function operates independently of the DBD. Validating the transcription self-activation activity of transcription factors is a critical step in ensuring the reliability of gene regulatory studies and protein interaction experiments. The yeast one-hybrid (Y1H) assay is commonly employed to detect the transcription self-activation activity of transcription factors. To ascertain the transcriptional activity, CmoPHR proteins were expressed in Y1HGold yeast strains. All transformants proliferated in SD/-Trp medium, confirming plasmid retention. Only CmoPHR2-BD-, CmoPHR9-BD-, CmoPHR16-BD-, and CmoPHR17-BD-expressing strains exhibited robust growth on transactivation-selective SD/-Trp/-His/-Ade medium (Figure 8), suggesting intrinsic transcriptional activation potential. CmoPHR1-BD and CmoPHR7-BD showed null activity, which is likely due to intramolecular repression domains within their full-length sequences.

## 3. Discussion

Recently, the *PHR* gene has been identified in some plants [22,28,31,32,33]. Although pumpkins are an important Cucurbitaceae crop, to our knowledge, there have been no studies on the *PHR* gene in pumpkin. In addition, no systematic studies on the *PHR* family genes in Cucurbitaceae have been reported. In this study, a total of 75 *PHR* genes were identified in six species of Cucurbitaceae, and phylogenetic analyses showed that they could be classified into seven major subfamilies (Group I, Group II, Group III, Group IV, Group V, Group VI, and Group VII), which is consistent with the conservation of *PHR* gene clustering in maize and rice (Figure 1), indicating that the functional differentiation of *PHR* genes in land plants has an ancient evolutionary origin [31]. Nine species of *PHR* genes appeared in the evolutionary clades. For example, in the clades belonging to groups I, III, IV, V, VI, and VII, each clade contained nine species of *PHR* genes. In Clade II, members of certain species disappeared and were found to contain members of cucumber, melon, watermelon, wax gourd, and pumpkin, but members of bottle gourd, rice, and corn were missing. Because each branch can be considered an ancestral *PHR* gene through replication and species differentiation, each branch represents copies and deletions specific to their gene lineage. It is of guiding significance to judge the evolution and origin of *PHR* gene family in Cucurbitaceae. The PHR transcription factor family integrates multiple signals to regulate plant development and environmental adaptation by activating or repressing thousands of genes through their specific sequences [22]. In our study, 11, 10, 8, 12, 12, and 22 PHR protein sequences were identified in cucumber, melon, bottle gourd, watermelon, wax gourd, and pumpkin, respectively (Table 1). The molecular weight range of all PHR proteins in the Cucurbitaceae is 25.51 to 83.31 kDa. It is noteworthy that all the identified PHR proteins may play conserved roles as phosphate-responsive proteins. It is noteworthy that pumpkin has significantly more *PHR* genes than other species (e.g., melon and bottle gourd), and it is possible that some genes were lost during the evolutionary process in other species. The remaining *PHR* genes appear to have great functional diversity, which is particularly important for plant adaptation to the environment. In addition, gene structure analysis showed that members of the same subfamily have a highly conserved exon–intron pattern. For example, exons of Group I genes are concentrated at the 3′ end, whereas those of Group II genes are concentrated at the 5′ end, and there are significant differences across subfamilies, suggesting that different subfamilies may adapt to diverse regulatory needs through selective splicing or structural variation (Figure 3C).

All the identified PHR proteins contain typical MYB-CC domains (Figure 3B), in which the MYB domain is responsible for binding the P1BS cis-element (GNATATNC), whereas the CC (coiled-coil) domain mediates homo- or heterodimerization, a feature that is highly conserved with the mechanism of function of *AtPHR1* in *Arabidopsis* [22]. However, the Cucurbitaceae PHR proteins, in addition to containing conserved motifs 1, 5, 3, and 2, also have species-specific motifs 6, 9, and 10 at the N- or C-terminus, which may confer a unique intercalating protein-binding ability or subcellular localization properties.

Promoter cis-regulatory elements determine the specific functions of genes [34]. The analysis of cis-regulatory elements can provide insights into gene expression and regulatory mechanisms in different tissues and stress environments. Several studies have reported the importance of ABRE, SARE, and MeJA in abiotic stress tolerance through plant hormone signaling pathways [1,35,36]. In the present study, the *PHR* gene of the cucurbit family contained many promoter cis-regulatory elements, such as ARE, ABRE, MeJA, GARE, SARE, and LTR, which are involved in light response, phytohormones, stresses, plant growth and development and so on(Figure 4), suggesting that PHR proteins may be integrated with other environmental stresses (e.g., drought, low-temperature stresses) to cross-regulate the phosphorus signaling network.. Notably, the co-occurrence of phosphorus-starvation-responsive motifs with drought/cold-stress elements suggests a PHR-mediated signal convergence—a meta-regulatory mechanism synchronizing Pi homeostasis with environmental stress adaptation. Crucially, this cis-regulatory architecture provides an evolutionary substrate for field-adaptive phosphorus-use efficiency in cucurbits, potentially enabling resource optimization under combinatorial abiotic stresses.

The expansion of plant gene families undergoes both genome-wide and tandem duplication, which leads to plant evolution and creates a diversity of gene functions [37,38]. Differences among the six Cucurbitaceae species may have been caused by genome-wide duplication events [39]. Seven fragment duplication events were identified in pumpkin, whereas a total of 1–2 fragment duplication events were identified in the other five species (Figure 5). This hypothesis is supported by our results. We also performed a covariance analysis for each of the six species (Figure 6). We speculate that they originated from the same ancestor. Therefore, these genes might have similar functions. These results provide a reference for the study of the PHR genes.

In plants, *PHR* genes are key transcription factors that respond to changes in soil phosphorus levels. In Arabidopsis, the four transcription factors of the Phosphate Starvation Response 1 (PHR1) family, namely PHR1 and its homologs PHR1-like 1 (PHL1), PHL2, and PHL3, form the central regulatory system that controls the expression of Pi-starvation-responsive (PSR) genes [40]. It has been shown that upregulation of the *PHR* gene enhances the ability of plants to absorb and utilize phosphorus when they experience phosphorus-starvation conditions, thereby promoting root growth and differentiation to improve phosphorus acquisition [22,27]. Through low-Pi- and high-Pi-stress experiments, this study found that pumpkin *CmoPHR1*, *CmoPHR3*, *CmoPHR9*, and *CmoPHR15* significantly responded to Pi starvation in the root system (Figure 7), and their expression levels were significantly upregulated after 4 weeks of low-Pi treatment but significantly suppressed under high-Pi conditions, suggesting that they may be involved in the regulation of phosphorus homeostasis and may participate in the phosphorus-deficiency signaling pathway to help plants adapt to a low-phosphorus environment by regulating the expression of related genes further downstream. This finding warrants further study to explore the specific functions and regulatory mechanisms of these genes in the phosphorus response. This is consistent with the regulatory pattern of rice *OsPHR2* [41,42], in which the SPX4 protein represses the activity of PHR2 by binding to it during phosphorus sufficiency, whereas the SPX4-PHR2 complex dissociates during phosphorus deprivation, releasing the transcriptional activation function of PHR. In addition, *CmoPHR2*, *CmoPHR7*, and *CmoPHR22* were highly expressed in the root system under both low- and high-Pi conditions, which may imply that they play a broader role in the perception and regulation of phosphorus in the root system. These genes might not only be involved in the response to phosphorus starvation but might also regulate the growth state of the root system when phosphorus is sufficient to prevent the accumulation of excess phosphorus or other nutrient imbalances. In addition, the expression patterns of these genes may be related to the efficiency of root uptake and utilization of other nutrients, suggesting their importance in plant nutrient homeostasis. *CmoPHR4*, *CmoPHR5*, *CmoPHR6*, *CmoPHR10*, *CmoPHR12*, *CmoPHR13*, *CmoPHR14*, *CmoPHR16*, and *CmoPHR18* in the root system responded significantly to high-Pi induction and showed different responses to other genes, suggesting that they may be involved in regulating root development or metabolic activities of plants in adapting to Pi-rich environments. In addition, the expression of *CmoPHR8* and *CmoPHR17* was significantly upregulated in roots treated with low-Pi for 2 weeks, suggesting that they could respond rapidly to low-phosphorus stress and may regulate the expression of downstream target genes. In pepper (*Capsicum annuum* L.), *CaPHR3* is rapidly upregulated in response to low-Pi stress, and overexpressing *CaPHR3* in *Arabidopsis thaliana* enhances Pi-starvation tolerance by modulating PSR-related genes [43]. The expression of *SmPHR7* in the *Arabidopsis thaliana* mutant *phr* (*SmPHR7*-OX) has been shown to partially rescue the phosphate-starvation phenotype. The expression of the Pi-starvation-induced (PSI) gene in *SmPHR7*-OX showed a significant induction compared with that of the *phr* mutant under phosphate starvation [33]. Furthermore, the expression of *CmoPHR21* was significantly upregulated in roots treated with high-Pi for 2 weeks. Some studies have found that the expression of *PHR* gene is not affected by low-Pi [2,44,45]. The upregulation of these genes may contribute to the enhancement of phosphorus uptake by the root system or the alteration of root growth patterns to adapt to nutrient-rich environments. Further experiments could reveal how these genes regulate morphological changes and physiological characteristics of the root system. Although the present study systematically investigated the transcriptional response patterns of pumpkin under sustained phosphorus stress through contrasting low-phosphate (2/4 weeks) and high-phosphate (2/4 weeks) treatments, which revealed gene expression differences between long-term phosphorus-starvation and phosphorus-replete states, the absence of “phosphate-level shift” experiments (e.g., transitioning from high- to low-phosphate conditions) limits our ability to directly capture the dynamic induction process for the PSR. Subsequent studies will employ time-course experiments involving a high-phosphate pre-acclimation phase followed by a low-phosphate shock to precisely dissect the dynamic regulatory network of the PSR and to validate the functions of key genes. Notably, the rapid response and sustained high expression of *PHR* genes in the root system of pumpkin, a typical representative of efficient phosphorus utilization in Cucurbitaceae (especially when used as a rootstock) [29,30], may synergize with its well-developed lateral root system and organic acid secretion capacity to enhance the activation and uptake efficiency of soil-insoluble phosphorus. These results emphasize the diversity of *CmoPHR* genes and their complexity in the adaptive response of the pumpkin root system to phosphorus utilization. This provides new insights into how plants adjust their growth under different nutrient conditions, and in-depth studies will help to reveal the mechanism of action of *PHR* genes in pumpkin and to provide a theoretical basis for improved nutrient management of these crops.

Since the PHR protein is a transcription factor, we verified the transcriptional activation activity of the CmoPHR proteins. The pGBKT7 vector and six ComPHR-pGBKT7 constructs were transformed into the Y1HGold yeast strain. Yeast assays showed that yeast cells containing CmoPHR2-BD, CmoPHR9-BD, CmoPHR16-BD, and CmoPHR17-BD expression vectors could grow normally on the defective SD/-Trp-His/-Ade medium, indicating that the gene had transcriptional self-activation activity (Figure 8). Wang et al. [46] found that AtPHL1 lacks transcription self-activation capability, whereas Zhou et al. [26] found that its closest homolog, OsPHR1/2, was self-activating. In our study, the closest homolog of AtPHL1, CmoPHR16, exhibits self-activity. It is possible that different species may have experienced different selection pressures during the process of evolution, leading to functional differentiation. The self-activation activity of CmoPHR2, CmoPHR9, CmoPHR16, and CmoPHR17 provides a new perspective for understanding the regulation of phosphorus signaling in pumpkin. A positive feedback mechanism may act as a “molecular switch” to amplify the stress response, and the functional differentiation suggests a complex regulatory network. In contrast, CmoPHR1 and CmoPHR7 showed null activity, which is likely due to intramolecular repression domains within their full-length sequences. This functional dichotomy implies that the transcriptional regulatory activity of CmoPHR proteins is governed by conformational plasticity, wherein N-terminal truncation or co-factor binding may mediate the transition from inactive to active isoforms by unlocking latent activation potential embedded within their repressive conformations.

## 4. Materials and Methods

### 4.1. Identification and Analysis of PHR Gene Family Members in Cucurbitaceae

The genome and predicted protein sequences of cucumber (*Cucumis sativus*; diploid, 2n = 14), melon (*Cucumis melo*; diploid, 2n = 24), bottle gourd (*Lagenaria siceraria*; diploid, 2n = 22), watermelon (*Citrullus lanatus*; diploid, 2n = 22), wax gourd (*Benincasa hispida*; diploid, 2n = 24), and pumpkin (*Cucurbita moschata*; allotetraploid, 2n = 40) were retrieved from the Cucurbitaceae genome database (http://cucurbitgenomics.org/; accessed on 18 January 2025). The genome and predicted protein sequences of maize and rice were obtained from the Phytozome database (https://phytozome-next.jgi.doe.gov/; accessed on 18 January 2025). Genome-wide identification of PHR-encoding genes in the six Cucurbitaceae species was conducted through a tripartite strategy. First, functionally characterized PHR proteins from maize (ZmPHR1) and rice (OsPHR1/OsPHR2) served as queries for homology-based searches against the proteomes of target species using BLASTp (E-value threshold: 1e-5) [47]. Second, Hidden Markov Model (HMM) profiles corresponding to the Myb_DNA-binding (PF00249) and Myb_CC_LHEQLE (PF14379) domains were retrieved from the Pfam database (http://pfam.xfam.org/; accessed on 19 January 2025) and subsequently applied to screen the Gramene database (http://www.gramene.org/; accessed on 19 January 2025) for these six Cucurbitaceae species. Third, domain validation of all non-redundant candidates was performed via manual curation using Pfam and SMART (http://smart.embl-heidelberg.de/; accessed on 19 January 2025) to confirm the presence of both conserved domains.

### 4.2. Physicochemical Properties and Chromosomal Localization Analysis

The coding sequence (CDS) length and chromosomal positions of the *PHR* genes were retrieved from the Cucurbitaceae genome database and visualized with TBtools (v2.149). For protein characterization, physicochemical parameters—including the relative molecular mass (Mw), isoelectric point (pI), and amino acid composition—were predicted using the ExPASy ProtParam tool (https://web.expasy.org/protparam/; accessed on 20 January 2025). Gene duplication events within the *PHR* family were further identified through collinearity analysis implemented in TBtools.

### 4.3. Phylogenetic Analysis of the PHR Gene Family in Cucurbitaceae

A comparative phylogenetic reconstruction of PHR proteins from Cucurbitaceae, maize, and rice was performed using the neighbor-joining (NJ) method in MEGA 11.0. The analysis incorporated 1000 bootstrap values with a Poisson substitution model to evaluate the branch support reliability. Topological visualization of the resultant phylogeny was optimized through the ChiPlot web platform (https://www.chiplot.online/#Phylogenetic-Tree; accessed on 21 January 2025) [48].

### 4.4. Gene Structure, Conserved Motifs, and Cis-Regulatory Element Prediction

The gene structures of the Cucurbitaceae PHR transcription factors were determined using TBtools (v2.149) based on genome annotations. Conserved protein motifs were characterized using the MEME Suite (http://meme-suite.org/tools/meme; accessed on 21 January 2025), with the motif count constrained to 10 and the remaining parameters at default values. Cis-regulatory elements within the 2000 bp promoter regions upstream of the PHR genes were interrogated through the PlantCARE database (https://bioinformatics.psb.ugent.be/webtools/plantcare/html/; accessed on 21 January 2025). All genomic features including the exon-intron organization, motif distribution, and regulatory elements were subsequently integrated and visualized using TBtools.

### 4.5. Plant Materials, Growth Conditions, and Treatments

Germinated pumpkin seedlings were hydroponically cultured in Hoagland nutrient solution under two phosphorus (Pi) gradient treatments: low-Pi (0.05 mmol·L^−1^) and high-Pi (2 mmol·L^−1^) regimes. Plants were maintained in a controlled greenhouse environment (28 °C; 16 h photoperiod/8 h dark cycle). Tissue sampling was performed at two developmental stages (2 and 4 weeks post-treatment), with leaves and roots collected separately. All samples were flash-frozen in liquid nitrogen and cryopreserved at −80 °C for downstream transcriptomic analysis.

### 4.6. RNA Isolation and qRT-PCR Analysis

Total RNA was extracted from pumpkin tissues using the FastPure Plant Total RNA Isolation Kit (RC401-01; Vazyme, Nanjing, China), followed by cDNA synthesis with HiScript^®^ III RT SuperMix (R323-01; Vazyme, Nanjing, China). Quantitative reverse-transcription PCR (qRT-PCR) was performed on a PikoReal Real-Time PCR System (TCR0096; Thermo Scientific, Waltham, MA, USA) with AceQ qPCR SYBR Green Master Mix (Q111-02; Vazyme, Nanjing, China), strictly adhering to the manufacturers’ protocols. The housekeeping gene *Cmoactin* (*actin* homolog) served as an internal control. Relative transcript abundance was calculated using the 2^−ΔΔCt^ method using triplicate biological replicates. Primer sequences are provided in Supplementary Table S1.

### 4.7. Gene Cloning, Recombinant Plasmid Assembly, and Transcriptional Activation Assay

Full-length *CmoPHR* cDNAs were PCR-amplified from pumpkin root RNA using gene-specific primers (Supplementary Table S2), with the resultant amplicons cloned into the TA/Blunt-Zero Vector (C601; Vazyme, Nanjing, China) for Sanger sequencing. To construct transcriptional activation reporters, termination-codon-truncated *CmoPHR* coding sequences (CDSs) were amplified (primers in Table S1) and directionally inserted into the pGBKT7 expression vector via *Nde* I/*EcoR* I restriction sites using homologous recombination cloning. Recombinant plasmid integrity was confirmed by restriction digestion and sequencing. For transcriptional activity screening, the empty pGBKT7 vector and the six CmoPHR-pGBKT7 constructs were individually transformed into Y1HGold yeast cells. Autonomous activation potential was assessed by monitoring yeast growth on SD/-Trp and SD/-Trp-His/-Ade selective media following established protocols [49].

## 5. Conclusions

In this study, 75 *PHR* genes were identified in six Cucurbitaceae species, with pumpkin (*Cucurbita moschata*) harboring the largest repertoire (22 members). A comprehensive study of the basic characteristics, chromosome location, gene structure, conserved motifs, promoters, and evolutionary relationships of these *PHR* genes has laid a solid foundation for understanding the functional diversification within the *PHR* gene family. Twenty *CmoPHR* genes showed differential expression patterns under different phosphorus treatments, indicating that *PHR* genes have specific functions in response to phosphate signals. Two kinds of CmoPHR3 and CmoPHR9 proteins were found to have self-activating activity. Our study establishes molecular blueprints for decoding PHR-mediated phosphorus homeostasis in Cucurbitaceae crops, with implications for developing nutrient-use-efficient varieties through targeted gene editing.

## Figures and Tables

**Table 1:** *PHR* genes identified in six Cucurbitaceae species.

Gene Name	Gene ID	Chromosome Location	Exon Number	CDS Length (bp)	Protein Length (aa)	MW (kDa)	pI
CsPHR1	CsGy1G031810.1	chr1:30837491-30843757 (−)	7	1389	462	51.36	5.14
CsPHR2	CsGy3G010900.1	chr3:8526938-8531213 (−)	6	933	310	33.65	6.02
CsPHR3	CsGy3G024970.1	chr3:25330062-25333810 (−)	8	1515	504	55.89	5.34
CsPHR4	CsGy3G026010.1	chr3:26761363-26766955 (−)	7	786	261	29.53	5.92
CsPHR5	CsGy3G034630.1	chr3:33313222-33318459 (+)	7	711	236	25.67	9.18
CsPHR6	CsGy3G038310.1	chr3:36043617-36047056 (+)	7	1191	396	43.01	8.85
CsPHR7	CsGy4G019000.1	chr4:25077536-25081462 (+)	7	1203	400	44.66	7.95
CsPHR8	CsGy6G011140.1	chr6:9566650-9569975 (−)	6	1092	363	40.00	8.86
CsPHR9	CsGy6G032210.1	chr6:29533121-29536937 (−)	7	840	279	30.90	8.93
CsPHR10	CsGy6G032220.1	chr6:29538118-29543334 (−)	6	969	322	35.20	6.56
CsPHR11	CsGy7G010430.1	chr7:13099901-13103260 (+)	8	1419	472	52.89	8.96
CmPHR1	MELO3C012590.2.1	chr01:20867349-20870178 (−)	7	1140	379	42.04	7.78
CmPHR2	MELO3C010134.2.1	chr02:14418218-14425559 (−)	8	1335	444	49.31	5.13
CmPHR3	MELO3C012848.2.1	chr04:13120451-13125000 (+)	8	1449	482	53.61	5.34
CmPHR4	MELO3C022765.2.1	chr04:21256362-21262033 (−)	8	933	310	34.79	8.28
CmPHR5	MELO3C026896.2.1	chr04:25859900-25862961 (−)	7	1215	404	43.83	8.85
CmPHR6	MELO3C009690.2.1	chr04:30135365-30141300 (−)	7	711	236	25.74	9.30
CmPHR7	MELO3C007988.2.1	chr08:6623374-6627144 (−)	8	813	270	29.67	8.71
CmPHR8	MELO3C007989.2.1	chr08:6628818-6634578 (−)	6	942	313	34.39	6.22
CmPHR9	MELO3C008929.2.1	chr08:23621008-23624170 (+)	7	1185	394	44.07	8.41
CmPHR10	MELO3C019621.2.1	chr11:25490228-25493530 (+)	6	1089	362	40.03	8.87
LsiPHR1	Lsi01G005190.1	chr01:4238229-4243824 (+)	6	942	313	34.44	6.56
LsiPHR2	Lsi01G005200.1	chr01:4247675-4251767 (+)	8	1032	343	38.03	6.93
LsiPHR3	Lsi03G012900.1	chr03:23709912-23713273 (−)	7	1137	379	41.49	7.73
LsiPHR4	Lsi05G010460.1	chr05:17974512-17988719 (−)	11	2238	745	83.31	6.49
LsiPHR5	Lsi05G018380.1	chr05:25663614-25670407 (+)	6	945	314	34.06	6.26
LsiPHR6	Lsi06G007750.1	chr06:15079402-15086717 (−)	9	1416	471	52.28	5.23
LsiPHR7	Lsi11G015860.1	chr11:24259033-24261452 (−)	6	1164	387	42.74	7.78
LsiPHR8	LsiUNG000820.1	chr00:2469195-2472643 (+)	7	783	260	29.38	8.61
ClaPHR1	ClCG01G021020.1	chr01:35006738-35010386 (−)	7	981	326	36.07	8.60
ClaPHR2	ClCG01G021030.1	chr01:35013406-35019457 (−)	6	969	322	35.39	6.38
ClaPHR3	ClCG02G018740.1	chr02:33478656-33486223 (+)	6	1080	359	39.60	9.03
ClaPHR4	ClCG03G009700.1	chr03:15190181-15199642 (+)	9	1611	536	59.49	5.62
ClaPHR5	ClCG04G002100.1	chr04:7040908-7046999 (−)	7	1197	398	44.75	6.52
ClaPHR6	ClCG05G003320.1	chr05:3139327-3143142 (−)	6	933	310	33.83	6.20
ClaPHR7	ClCG07G001320.1	chr07:1402922-1406979 (−)	7	1452	483	53.53	5.32
ClaPHR8	ClCG07G002310.1	chr07:2352358-2356712 (+)	9	918	305	34.20	6.05
ClaPHR9	ClCG09G022860.1	chr09:39854276-39856296 (+)	6	1164	387	42.77	7.30
ClaPHR10	ClCG10G012620.1	chr10:26664138-26666981 (−)	7	846	282	33.14	6.34
ClaPHR11	ClCG10G013290.1	chr10:27482619-27485716 (−)	7	1149	382	42.07	8.43
ClaPHR12	ClCG11G007240.1	chr11:8067404-8068916 (+)	5	891	296	33.60	6.06
BhiPHR1	Bhi01M000162	chr1:4358307-4374348 (−)	17	1734	577	5.94	64.66
BhiPHR2	Bhi02M001052	chr2:30073057-30079744 (+)	8	1341	446	5.32	49.72
BhiPHR3	Bhi03M000545	chr3:13712964-13718673 (+)	6	942	313	6.60	34.44
BhiPHR4	Bhi03M000547	chr3:13774321-13778870 (+)	7	978	325	7.77	35.90
BhiPHR5	Bhi03M002344	chr3:72814246-72818856 (−)	7	1155	384	6.52	43.24
BhiPHR6	Bhi05M000006	chr5:339928-345085 (+)	7	783	260	6.47	29.13
BhiPHR7	Bhi05M000125	chr5:3492888-3498142 (+)	8	1449	482	5.39	53.54
BhiPHR8	Bhi05M001707	chr5:61216044-61218783 (−)	7	1149	382	7.78	41.95
BhiPHR9	Bhi06M000772	chr6:23276891-23278379 (−)	6	753	251	8.20	28.35
BhiPHR10	Bhi10M000727	chr10:19061719-19064970 (−)	6	1095	364	8.87	39.99
BhiPHR11	Bhi11M001288	chr11:43469320-43472785 (−)	7	1143	380	8.43	41.69
BhiPHR12	Bhi11M002259	chr11:73208843-73217031 (+)	7	711	236	9.37	25.80
CmoPHR1	CmoCh01G005130	chr01:2468014-2469314 (+)	4	666	221	25.51	8.91
CmoPHR2	CmoCh02G016940	chr02:9712038-9714929 (+)	6	723	240	27.29	8.41
CmoPHR3	CmoCh02G017700	chr02:10102355-10106076 (+)	8	1314	437	48.55	5.13
CmoPHR4	CmoCh03G000340	chr03:656888-659538 (−)	7	1131	376	42.11	6.16
CmoPHR5	CmoCh03G006380	chr03:5845977-5850993 (+)	7	1095	364	40.86	6.43
CmoPHR6	CmoCh03G006390	chr03:5852404-5856186 (+)	7	984	327	36.12	7.17
CmoPHR7	CmoCh05G008680	chr05:5707748-5709145 (+)	5	756	251	28.19	6.64
CmoPHR8	CmoCh06G000560	chr06:349595-359369 (−)	16	1803	600	67.55	9.25
CmoPHR9	CmoCh06G012530	chr06:9468323-9472100 (−)	6	951	316	34.29	6.09
CmoPHR10	CmoCh06G017240	chr06:11843093-11845080 (−)	6	1152	383	41.82	8.69
CmoPHR11	CmoCh07G003670	chr07:1675620-1680532 (+)	8	1086	361	39.81	8.43
CmoPHR12	CmoCh07G003680	chr07:1680538-1683042 (+)	6	972	323	35.51	8.30
CmoPHR13	CmoCh07G012910	chr07:7134301-7137195 (−)	7	1155	384	43.29	6.11
CmoPHR14	CmoCh10G010790	chr10:5854637-5859651 (+)	9	1356	451	50.19	5.31
CmoPHR15	CmoCh10G012730	chr10:13133524-13137613 (+)	5	1191	396	44.27	8.27
CmoPHR16	CmoCh11G009860	chr11:5250875-5255809 (+)	8	1326	441	49.06	5.09
CmoPHR17	CmoCh14G011540	chr14:9041919-9044422 (+)	6	1152	383	41.95	8.16
CmoPHR18	CmoCh14G019460	chr14:14393688-14400483 (+)	8	1071	356	38.47	6.04
CmoPHR19	CmoCh19G001230	chr19:723628-727308 (−)	7	1167	388	43.63	5.43
CmoPHR20	CmoCh19G002200	chr19:1459343-1470768 (−)	9	783	260	29.29	6.17
CmoPHR21	CmoCh19G011500	chr19:9659420-9662090 (+)	6	1140	379	41.48	7.75
CmoPHR22	CmoCh20G003120	chr20:1530207-1533405 (+)	6	1101	366	40.43	8.87

**Table 2:** The homologous *PHR* gene pairs between pumpkin and other Cucurbitaceae species.

Pumpkin	Melon	Watermelon	Cucumber	Wax Gourd	Bottle Gourd
*CmoPHR1*	-	-	-	*BhiPHR9*	-
*CmoPHR2*	*CmPHR4*	*ClaPHR8*	*CsPHR4*	*BhiPHR6*	*LsiPHR8*
*CmoPHR3*	*CmPHR3*	*ClaPHR7/ClaPHR19*	*CsPHR3*	*BhiPHR7*	-
*CmoPHR4*	*CmPHR9*	-	*CsPHR7*	*BhiPHR5*	-
*CmoPHR5*	*CmPHR7*	*ClaPHR1*	*CsPHR10*	*BhiPHR3*	*LsiPHR1*
*CmoPHR6*	-	-	*CsPHR9*	-	-
*CmoPHR7*	-	*ClaPHR12*	-	-	-
*CmoPHR8*	-	-	*CsPHR5*	*BhiPHR12*	-
*CmoPHR9*	-	*ClaPHR6*	*CsPHR2*	-	*LsiPHR5*
*CmoPHR10*	*CmPHR5*	*ClaPHR11*	*CsPHR6*	*BhiPHR11*	*LsiPHR3*
*CmoPHR11*	*CmPHR7*	*ClaPHR1*	*CsPHR10*	*BhiPHR3*	*LsiPHR1*
*CmoPHR12*	-	-	*CsPHR9*	-	-
*CmoPHR13*	*CmPHR9*	-	*CsPHR7*	*BhiPHR5*	-
*CmoPHR14*	*CmPHR2*	-	*CsPHR1*	*BhiPHR2*	*LsiPHR6*
*CmoPHR16*	*CmPHR2*	-	-	-	-
*CmoPHR18*	-	*ClaPHR6*	*CsPHR2*	*BhiPHR1*	*LsiPHR5*
*CmoPHR19*	*CmPHR3*	-	*CsPHR3*	*BhiPHR7*	*LsiPHR4*
*CmoPHR20*	*CmPHR4*	*ClaPHR8*	*CsPHR4*	*BhiPHR6*	*LsiPHR8*
*CmoPHR21*	*CmPHR1*/*CmPHR10*	*ClaPHR3*/*ClaPHR9*	*CsPHR8*/*CsPHR11*	*BhiPHR8*/*BhiPHR10*	*LsiPHR7*
*CmoPHR22*	*CmPHR1*/*CmPHR10*	*ClaPHR3*/*ClaPHR9*	*CsPHR8*	*BhiPHR8*/*BhiPHR10*	*LsiPHR7*

## Data Availability

The data presented in this study are available in the article or Supplementary Materials.

## Supplementary Materials

The following supporting information can be downloaded at: https://www.mdpi.com/article/10.3390/plants14101443/s1. Table S1. Primers for qRT-PCR. Table S2. Primers for gene cloning and vector construction.
