# Supplementary material for: Genome-Wide Identification of the PHR Gene Family in Six Cucurbitaceae Species and Its Expression Analysis in Cucurbita moschata
Source: Plants (Basel). 2025 May 12;14(10):1443. doi: 10.3390/plants14101443 (PMC12115016; doi:10.3390/plants14101443)
Supplement: Supplementary file 1 [file plants-14-01443-s001.zip › plants-3559728-supplementary.pdf]

Table S1. Primers for qRT-PCR.

| Primer name | Primer Sequence |
|---|---|
| q*Cmoβ-Actin*-F | GTGCCTGCTATGTATGTTGCC |
| q*Cmoβ-Actin*-R | GGTCCAAACGGAGAATGGCATG |
| q*CmoPHR1*-F | GGAAGACCCAGAAGCCTGAA |
| q*CmoPHR1*-R | CCAGCGCTTCAGAACAACAC |
| q*CmoPHR2*-F | AGCACAAAAGAGTCTGGCACT |
| q*CmoPHR2*-R | ATCGTTTCCATTTGCCGCAC |
| q*CmoPHR3*-F | AGTAGTGCAGAGGTTTCAATGG |
| q*CmoPHR3*-R | ACGAAACGGATGAGTAGTGAAGA |
| q*CmoPHR4-F* | CAGCTCGATGTTCAGAGGCG |
| q*CmoPHR4-R* | CGACGGGTCGTTAGGGAAC |
| q*CmoPHR5-F* | GGAGGAATCGTCGTCGTTTAT |
| q*CmoPHR5-R* | CATCGGGATTAACAAGCCTCG |
| q*CmoPHR6-F* | TCGAACATTGAGGGCTCGTC |
| q*CmoPHR6-R* | AGCTGCTCCTGAAAGAACCC |
| q*CmoPHR7-F* | CACTGGGCTAAGGCTGTACC |
| q*CmoPHR7-R* | CATCGCTGTTTTCCACGTCC |
| q*CmoPHR8-F* | TCGAATGGTAGTGTCGTCGTC |
| q*CmoPHR8-R* | TGAGAGTCGAGTCCAGGGTT |
| q*CmoPHR9*-F | CCAGAAATATCGGCTCGGGA |
| q*CmoPHR9*-R | CCGTGACTTGGAAACCGTCG |
| q*CmoPHR10*-F | AAGCTGTGAACCAGCTTGGA |
| q*CmoPHR10*-R | CACTGTCCCTACACGCCAA |
| q*CmoPHR11*-F | TATCCGAGTGCGACATGCAA |
| q*CmoPHR11*-R | TATTGGGCGCCACTTTCTGA |
| q*CmoPHR12*-F | GTGCACAAGTGGTTCACTCT |
| q*CmoPHR12*-R | CGCTTCTGGACCTCCATCTG |
| q*CmoPHR13*-F | CAGCTAGATGTTCAGAGGCGT |
| q*CmoPHR13*-R | GGGTCGTCGGAGTTGTTGAA |
| q*CmoPHR14-F* | GCAAAGCATTGCTGGTGTGT |
| q*CmoPHR14-R* | TTGAGCAAGTCCATGTCGTT |
| q*CmoPHR15-F* | AGCAGCCTCACAAGGAGTTC |
| q*CmoPHR15-R* | CCTGAAGCTGAATCAAGAGCG |
| q*CmoPHR16-F* | TCCGAAGCCATTTGAAGAGAAG |

| | |
|---|---|
| q*CmoPHR16-R* | GACAACGGTTGCATCAATGG |
| q*CmoPHR17-F* | GCACCATGAATGAGATGCAAA |
| q*CmoPHR17-R* | CCATGTTCTCACCCGCAAG |
| q*CmoPHR18-F* | AGGCCACTCCTAAAACCGTG |
| q*CmoPHR18-R* | TACACGCCAACCCATACCAG |
| q*CmoPHR19*-F | CCACCGGAATGAAGAGACCG |
| q*CmoPHR19*-R | ACAAATGGAACGGGTTTCGC |
| q*CmoPHR20*-F | CATCACAGAGGCTCTACGGC |
| q*CmoPHR20*-R | TGGATCGGTGAATCGTGTGG |
| q*CmoPHR21*-F | AACTCGGTGGCCCTGAAAAA |
| q*CmoPHR21*-R | GAAGTTGCTCAGAGTCCCACT |
| q*CmoPHR22*-F | TATTTACCTCCGAACCGCCG |
| q*CmoPHR22*-R | GCGGTACTAAGGTGGCTGAA |

Table S2. Primers for gene cloning and vector construction.

| **Primer name** | **Primer Sequence** |
|---|---|
| *CmoPHR1*-F | ATGGATATTCAAAGAGAAAAAGA |
| *CmoPHR1*-R | TCAAACATACTCCACATTCAAAT |
| *CmoPHR2*-F | ATGGAACGGAATTATCCGTAT |
| *CmoPHR2*-R | AAGCCACCTTTTGGATTTCA |
| *CmoPHR7*-F | ATGCAGAATCATCACCACATCAAC |
| *CmoPHR7*-R | TCAAAGAAACTCAATCAACTCTGATC |
| *CmoPHR9-F* | ATGCTCTCTGGGTTTTCTC |
| *CmoPHR9-R* | TCATCCAATATTACTCATCATCC |
| *CmoPHR16-F* | ATGTCATCATCGTATCAAGTTCTTC |
| *CmoPHR16-R* | CTATAAACCTGTTGCATCTGATT |
| *CmoPHR17-F* | ATGTTCCATCCTAATAAGAAAGCT |
| *CmoPHR17-R* | TTAACCGAAGGGTGAATTTG |
| BD-*CmoPHR1*-F | tcagaggaggacctgcatatgATGGATATTCAAAGAGAAAAAG |
| BD-*CmoPHR1*-R | tcgacggatccccgggaattcAACATACTCCACATTCAAATCGATG |
| BD-*CmoPHR2*-F | tcagaggaggacctgcatatgATGGAACGGAATTATCCGTAT |
| BD-*CmoPHR2*-R | tcgacggatccccgggaattcAAGCCACCTTTTGGATTTC |
| BD-*CmoPHR7*-F | tcagaggaggacctgcatatgATGCAGAATCATCACCAC |
| BD-*CmoPHR7*-R | tcgacggatccccgggaattcTCAAAGAAACTCAATCAACTC |

| | |
|---|---|
| BD-*CmoPHR9-F* | tcagaggaggacctgcatatgATGCTCTCTGGGTTTTCTCAGG |
| BD-*CmoPHR9-R* | tcgacggatccccgggaattcTCCAATATTACTCATCATCCATTCG |
| BD-*CmoPHR16-F* | tcagaggaggacctgcatatg ATGTCATCATCGTATCAAGTTC |
| BD-*CmoPHR16-R* | tcgacggatccccgggaattc CTATAAACCTGTTGCATCTGA |
| BD-*CmoPHR17-F* | tcagaggaggacctgcatatgATGTTCCATCCTAATAAGAAAGCTTC |
| BD-*CmoPHR17-R* | tcgacggatccccgggaattcACCGAAGGGTGAATTTGTGGT |
